# Die Medizininformatik-Initiative und Seltene Erkrankungen: Routinedaten der nächsten Generation für Diagnose, Therapiewahl und Forschung

**DOI:** 10.1007/s00103-022-03606-y

**Published:** 2022-10-28

**Authors:** Josef Schepers, Julia Fleck, Jannik Schaaf

**Affiliations:** 1 Berlin Institute of Health (BIH) at Charité – Universitätsmedizin Berlin, Anna-Louisa-Karsch Str. 2, 10178 Berlin, Deutschland; 2 Zentrum für Seltene Erkrankungen, Universitätsklinikum Aachen, Aachen, Deutschland; 3 Institut für Medizininformatik, Universitätsklinikum Frankfurt am Main, Goethe Universität Frankfurt, Frankfurt am Main, Deutschland

**Keywords:** Standardisierte Gesundheitsdaten, Interoperabilität, Vernetzung von Versorgung und Forschung, CORD-MI, Waisenkinder der Medizin, Standardised health data, Interoperability, Digital integration of care and research, CORD-MI, Orphan diseases

## Abstract

Bei Menschen mit Seltenen Erkrankungen (SE) besteht ein besonderes Potenzial, von der Digitalisierung im Gesundheitswesen zu profitieren. Das Nationale Aktionsbündnis für Menschen mit Seltenen Erkrankungen (NAMSE) hat sich dafür eingesetzt, dass SE bei der Digitalisierung des Gesundheitswesens in Deutschland konkret berücksichtigt werden. In der Medizininformatik-Initiative (MII) des Bundesministeriums für Bildung und Forschung (BMBF) wurde das Thema aufgegriffen. Hier wird aktuell ausgehend von den Universitätskliniken eine digitale Infrastruktur für die datenschutzkonforme Mehrfachnutzung von standardisierten Versorgungs- und Forschungsdaten aufgebaut. Teil der Initiative ist seit dem Jahr 2020 das Projekt CORD-MI (Collaboration on Rare Diseases), in dem sich Universitätskliniken und weitere Partner deutschlandweit zusammengeschlossen haben, um die Patientenversorgung und die Forschung im Bereich der SE zu verbessern.

In diesem Beitrag wird beleuchtet, in welcher Weise die MII die Belange der SE berücksichtigt und welche Chancen die gewonnenen „neuen Routinedaten“ bieten. Ein SE-Modul wurde in den „MII-Kerndatensatz“ aufgenommen – ein Informationsmodell, das auf dem Datenstandard „FHIR“ (Fast Healthcare Interoperability Resources) basiert. Daten, die im Rahmen von Versorgungs- und Forschungsroutinen erhoben werden, können so zukünftig zwischen den beteiligten Einrichtungen ausgetauscht werden und im Bereich SE z. B. die Diagnosefindung, die Therapiewahl und Forschungsvorhaben unterstützen. Das Projekt CORD-MI hat sich zum Ziel gesetzt, mit Hilfe exemplarischer Fragestellungen Erkenntnisse über die Versorgungssituation von Menschen mit SE zu erhalten und daraufhin Rückschlüsse für weitere notwendige Schritte im Bereich der Digitalisierung zu ziehen.

## Abstract (en)

People with rare diseases (RDs) have particular potential to benefit from digitisation in the healthcare system. The National Action Alliance for People with Rare Diseases (NAMSE) has campaigned for SE to be specifically taken into account in the digitisation of the healthcare system in Germany. The topic was addressed within the Medical Informatics Initiative (MII) of the Federal Ministry of Education and Research (BMBF). Here, starting with university hospitals, a digital infrastructure is currently being established for the data protection-compliant multiple use of standardised care and research data. Since 2020, part of the initiative has been the CORD-MI project (Collaboration on Rare Diseases) in which university hospitals and other partners throughout Germany have joined forces to improve patient care and research in the field of rare diseases.

This article highlights how the MII takes into account the concerns of SE and what opportunities the “new routine data” obtained offer. A SE module was included in the “MII core data set” – an information model based on the data standard fast healthcare interoperability resources (FHIR). Data collected in the context of care and research routines can thus be exchanged between the participating institutions in the future and support, for example, diagnosis, therapy selection and research projects in the field of SE. The CORD-MI project has set itself the goal of obtaining insights into the care situation of people with SE with the help of exemplary questions and then drawing conclusions for further necessary steps in the area of digitalisation.

## Einleitung

Damit die Digitalisierung auch für die Menschen mit Seltenen Erkrankungen (SE)^1^ von Nutzen ist, bedarf es verschiedener Maßnahmen, bei denen die Gegebenheiten des deutschen Gesundheitssystems, aber auch die Besonderheiten der SE berücksichtigt werden müssen. Das deutsche Gesundheitssystem weist sehr ausgeprägt gegliederte Versorgungsstrukturen und entsprechend vielseitige Sammlungen von Daten, sogenannte Datensilos, auf. Dies hat bisher die vollständige Zusammenstellung von Gesundheits‑, Erkrankungs- und genetischen Daten einzelner Personen erschwert und merklich die konsistente Bildung von effizient auswertbaren Datenbeständen für Gruppen von Betroffenen behindert. Gerade für SE, bei denen die Daten meist in verschiedenen medizinischen Einrichtungen erfasst werden, sind die Zusammenführung von Daten und deren aussagekräftige Auswertung für Diagnostik- und Therapieerfolge unerlässlich.

Denn mit der Seltenheit der SE, dem fehlenden Überblick über die Verbreitung der einzelnen Erkrankungen sowie der Schwierigkeit, diagnostische und therapeutische Studiengruppen zusammenzustellen, sind große Herausforderungen in diesem Bereich markiert. Um die Situation grundsätzlich zu verbessern, ist im Jahr 2009 von den Bundesministerien für Bildung und Forschung sowie Gesundheit (BMBF und BMG) und von der Patientendachorganisation Allianz Chronischer Seltener Erkrankungen (ACHSE e. V.) das Nationale Aktionsbündnis für Menschen mit Seltenen Erkrankungen (NAMSE; [1]) gegründet worden. Dort sind alle relevanten Stakeholder des Gesundheitssystems (28 Bündnispartner) vertreten.

Infolge der deutschen NAMSE-Aktivitäten, die die Voraussetzung für die deutsche Beteiligung an den europäischen Anstrengungen sind, entstanden im Laufe der 2010er-Jahre in fast allen deutschen Universitätskrankenhäusern zentrale Anlaufstellen für die Versorgung und Erforschung der SE: die Zentren für Seltene Erkrankungen (ZSE). Die ZSE haben verschiedene Erkrankungsexpertisen und sind in der Arbeitsgemeinschaft der ZSE organisatorisch miteinander vernetzt. Vernetzung und Abstimmung sind unter anderem in den Innovationsfondsprojekten TRANSLATE-NAMSE^2^ und ZSE DUO^3^ vorangebracht worden. Es wurde deutlich gezeigt, was im Bereich der SE erreicht werden kann, wenn Diagnose und Versorgungswege interdisziplinär konzipiert und strukturiert angelegt werden. Es ist zugleich nochmals deutlich geworden, dass für weitere Verbesserungen eine gemeinsame digitale Infrastruktur und präzise, abgestimmte Dokumentationen in den ZSE benötigt werden.

Angeregt durch das NAMSE wurde in der Medizininformatik-Initiative (MII) des Bundesministeriums für Forschung und Bildung (BMBF) das Thema der SE aufgegriffen [2]. In der MII wird aktuell ausgehend von den Universitätskliniken eine digitale Infrastruktur für die datenschutzkonforme Mehrfachnutzung von standardisierten Versorgungs- und Forschungsdaten aufgebaut. Teil der Initiative ist seit dem Jahr 2020 das Projekt CORD-MI (Collaboration on Rare Diseases; [14]), in dem sich Universitätskliniken und weitere Partner deutschlandweit zusammengeschlossen haben, um die Patientenversorgung und die Forschung im Bereich der SE zu verbessern.

Die „länderübergreifende Vernetzung“ [2] ist im Bereich der SE, insbesondere der ultraseltenen Erkrankungen, eine notwendige Ergänzung der nationalen Zusammenarbeit. Daher erfolgt im Vorhaben CORD-MI insbesondere die Abstimmung mit den Europäischen Referenz-Netzwerken (ERN), mit der Europäische Plattform für die Registrierung Seltener Krankheiten (EU RD Platform) sowie mit dem Netzwerk Orphanet, das ein Portal für seltene Krankheiten und deren Therapeutika, die „Orphan Drugs“, betreibt. Darüber hinaus soll mit der „Global Alliance for Genomics and Health“ (GA4GH) der Erfahrungsaustausch über die Verwendung von „Phenopackets“^4^ [3–5] bei der standardisierten Phänotypisierung fortgesetzt werden.

Ziel des vorliegenden Beitrags ist es, die deutsche Medizininformatik-Initiative vorzustellen und speziell die Integration des Bereichs der SE zu beschreiben. Der Schwerpunkt der Betrachtung liegt auf den Anstrengungen für die standardisierte Verfügbarkeit von aufbereiteten Versorgungs- und Forschungsdaten. Die Chancen, welche durch die „neuen Routinedaten“ für SE entstehen, werden aufgezeigt.

## Die Medizininformatik-Initiative – Zusammenarbeit für Seltene Erkrankungen

Im deutschen Gesundheitssystem gibt es rund 200.000 Arztpraxen und 2000 Krankenhäuser, wobei fast jede Einrichtung in Kombination mit Papierakten eigene IT-Systeme betreibt, zwischen denen bisher nur in beschränktem Umfang eine gemeinsame Datenverarbeitung stattfindet. Eine gemeinsame, standortübergreifende Datennutzung wäre aber – insbesondere im Bereich der SE – dringend geboten.

Zur Bewältigung dieser Herausforderungen und zur Demonstration von Lösungen hat das BMBF in der Mitte der 2010er-Jahre das längerfristige Förderprogramm Medizininformatik-Initiative (MII; [6–8]) aufgelegt mit dem Schwerpunkt in den Universitätskliniken. Versorgungs- und Forschungsdaten, die bei vielen Anwendungen und in vielen lokalen Bereichen zunehmend zahlreicher werden, aber oft getrennt und heterogen entstehen, sollen durch eine einrichtungsübergreifende gemeinsame Nutzung auf der Basis eines abgestimmten Informationsmodells [6–8] aus Krankenversorgung und Forschung besser nutzbar gemacht werden. Besondere Beachtung soll die IT-basierte Unterstützung von Diagnose und Therapiewahl bei Seltenen Erkrankungen finden [9].

Die Fördermaßnahme soll die medizinische Forschung stärken und die Patientenversorgung verbessern, insbesondere indem Erkenntnisse aus der Forschung beschleunigt die Patientinnen und Patienten erreichen. Diese Zielsetzung passt zu dem vom NAMSE in der NAMSE-Strategie 2020 bis 2022 angezeigten Digitalisierungsbedarf für Menschen mit SE.

Kernelemente der MII sind die „lokalen Datenintegrationszentren an allen teilnehmenden Standorten“ und „IT-Lösungen für Anwendungsfälle“, zu denen im Rahmen der Zusammenarbeit im Programm als drittes Kernelement noch das zentrale „Deutsche Forschungsdatenportal Gesundheit“ (FDP-G) hinzugekommen ist.

Im Jahr 2018 haben die 4 Primärkonsortien DIFUTURE, HIGHmed, MIRACUM und SMITH [10], die zusammen inzwischen fast alle deutschen Universitätskliniken umfassen, sich dieser Aufgaben angenommen. Im Laufe des Jahres 2020 sind in Form von Verbundvorhaben mit 13 bis 24 beteiligten Standorten ergänzend die 3 Primärkonsortien-übergreifenden Verbundvorhaben CORD-MI, POLAR-MI und ABIDE-MI [10] hinzugekommen, die die Datenaufbereitung aller im Aufbau befindlichen Datenintegrationszentren (DIZ) nutzen und darauf eigene Anwendungen (Use Cases) aufsetzen. Bei CORD-MI stehen SE im Fokus.

### Erstes Kernelement der MII: Datenintegrationszentren.

Die an jedem Standort aufgebauten DIZ [11] haben die Aufgabe, ein großes Spektrum der Versorgungs- und Forschungsdaten ihrer Standorte organisatorisch und technisch aufzuarbeiten, so dass diese sowohl für einrichtungsinterne als auch für einrichtungsübergreifende Mehrfachnutzungen datenschutzkonform zur Verfügung stehen. Das zentrale Element für die Ermöglichung der gemeinsamen Datennutzung ist das gemeinsame Informationsmodell der MII, der MII-Kerndatensatz, nach dem alle DIZ ihre Datenaufbereitung ausrichten. In der aktuell laufenden Aufbau- und Vernetzungsphase (2018–2022) haben sich alle Standorte zunächst auf die Aufbereitung der Versorgungsdaten aus ihren klinischen Informationssystemen konzentriert. Insbesondere im Bereich der SE sind jedoch weitere abgestimmte Dokumentationsformen im Versorgungs- und im Forschungskontext vonnöten und werden unter Beachtung des um ein SE-Modul ergänzten Informationsmodells entwickelt.

**Figure 1:**
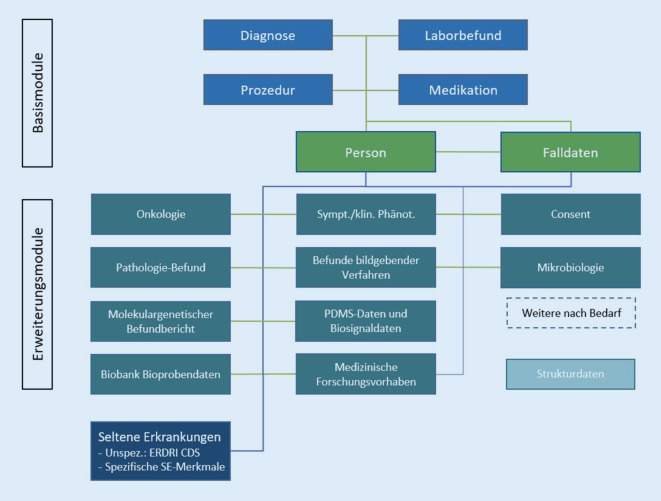

In Abb. 1 ist das Schema des MII-Kerndatensatzes skizziert. Es werden Basis- und Erweiterungsmodule unterschieden, wobei das SE-Modul zu den Erweiterungsmodulen zählt. Eine genauere Beschreibung der nach wie vor beweglichen Module findet sich auf der Homepage der MII. Die Datenaufbereitung aus den Quellsystemen erfolgt in den DIZ aller Primärkonsortien und damit an allen Standorten der MII synchronisiert. Entscheidend für den Erfolg der MII ist die einheitliche Bereitstellung in allen Kliniken. Daran können sich verschiedene Nutzungsmöglichkeiten anschließen. Sinnvoll ist es, bei Neuanschaffungen und anderen IT-Anpassungen das Informationsmodell des MII-Kerndatensatzes schon in den Quellsystemen zu implementieren.

### Zweites Kernelement der MII: IT-Lösungen für spezifische Anwendungen.

Das zweite Kernelement der MII ist die Entwicklung von „IT-Lösungen für spezifische Anwendungen, für die der standortübergreifende Austausch von Forschungs- und Versorgungsdaten genutzt werden soll“ [12]. Neben dem Aufbau der DIZ sind die 4 Primärkonsortien seit 2018 innerhalb ihrer Verbünde, die aus 5–10 Klinikstandorten und weiteren Partnereinrichtungen bestehen, mit Use Cases in verschiedenen medizinischen Fachdisziplinen wie Onkologie, Intensivmedizin, Infektiologie, Neurologie oder Kardiologie befasst [13]. Der Verbund CORD-MI [14] bereitet durch die Umsetzung einfacher Beobachtungsstudien zu ausgewählten SE die einrichtungsübergreifende „IT-basierte Unterstützung von Diagnosefindung und Therapiewahl bei Seltenen Erkrankungen“ in der nächsten Phase der MII vor.

### Drittes Kernelement der MII: das Deutsche Forschungsdatenportal für Gesundheit (FDP-G).

Damit Wissenschaftlerinnen und Wissenschaftler sowie interessierte Patientengruppen nicht alle Standorte einzeln ansprechen müssen, um Daten für ein neues Forschungsprojekt zu beantragen, wird als verbindendes drittes Kernelement der MII das „Deutsche Forschungsdatenportal für Gesundheit“ (FDP‑G; [15]) als zentraler Ansprechpartner und Organisator aufgebaut. Das Portal unterstützt das verantwortungs- und datenschutzkonforme Paradigma der MII, wonach die universitätsmedizinischen Standorte ihre Daten dezentral halten und mit Hilfe ihrer lokalen „Data Use & Access Committees“ (DUAC/UAC) auch die Hoheit darüber behalten. Mit Hilfe ihrer DUAC entscheiden die Häuser, an welchen Forschungsprojekten sie sich mit den Daten ihrer Patientinnen und Patienten beteiligen möchten. Entsprechende Anfragen werden über das FDP‑G vermittelt.

## Die nächste Generation von Routinedaten

Das gemeinsame Informationsmodell der MII, der „MII-Kerndatensatz“ (MII-KDS), basiert auf dem Standard für den Datenaustausch im Gesundheitswesen „FHIR“ (Fast Healthcare Interoperability Resources, gesprochen wie engl. „fire“), an dem die Krankenhäuser ihre IT-Entwicklungen anpassen und alle DIZ ihre Datenaufbereitungen ausrichten. Es spielt eine zentrale Rolle bei der gemeinsamen Datennutzung der Universitätskliniken. Mit diesem Informationsmodell wird sowohl der vom NAMSE geforderte standardisierte „Austausch von Patienten- und Forschungsdaten“ als auch die abgestimmte, längerfristige Speicherung in datenschutzkonformen lokalen Datenbanken („geschützten Räumen“) für multizentrische Analysen der national und international vernetzten Partnereinrichtungen unterstützt. Und obwohl die Entwicklung noch nicht abgeschlossen und die Umsetzung noch auf die Universitätskliniken begrenzt ist, werden die neuen Datenbestände – zumindest im Kreis der Beteiligten – bereits als die „nächste Generation von Routinedaten“ bezeichnet.

Unter Routinedaten werden hier Gesundheits‑, Krankheits-, genetische und Prozessdaten im Zeitverlauf verstanden, die im Rahmen von Versorgungsprozessen routiniert erhoben werden und auf der Basis standardisierter Bereitstellung in Auswertungsprozessen routinemäßig genutzt werden können. Die neue Generation der Routinedaten wird von sogenannten Fach- und Organspezifischen Arbeitsgruppen (FOSA) bestimmt. Im Schema des MII-Kerndatensatzes (Abb. 1) ist erkennbar, dass dieses Informationsmodell sich nicht auf Einrichtungs- und Abteilungskontakte sowie Diagnosen und Prozeduren beschränkt, sondern viele medizinische Merkmale berücksichtigt. Hervorgehoben werden können Laborbefunde, Medikationen, Symptome/Phänotypen und molekulargenetische Befundberichte. Zudem beschränkt sich das Modell bei Diagnosen und Prozeduren nicht auf Kodierungen durch ICD^5^- und OPS^6^-Codes, sondern empfiehlt die ergänzende Verwendung von Orpha-Kennnummern^7^ und SNOMED-CT^8^-Termen. Dabei ist die Einbeziehung von Merkmalen nicht auf die in den Informationssystemen vorhandenen Felder beschränkt, vielmehr können durch die FOSA-abgestimmte Gestaltung der Inhalte und der Formen der Erweiterungsmodule des MII-Kerndatensatzes neue Datenerhebungen entwickelt werden, ohne dass von allen Parteien derselbe IT-Lieferant beauftragt werden muss.

Gestärkt wird die Umsetzung des MII-KDS durch das Zusammenwirken mit den Standardisierungsaktivitäten der Kassenärztlichen Bundesvereinigung (KBV) im Bereich der Medizinischen Informationsobjekte (MIO; [16]), der Nationalen Agentur für Digitale Medizin (gematik) zu Informationstechnischen Systemen in Krankenhäusern (ISiK; [17]) und weiterer Akteure mit weiteren Ansätzen.

**Figure 2:**
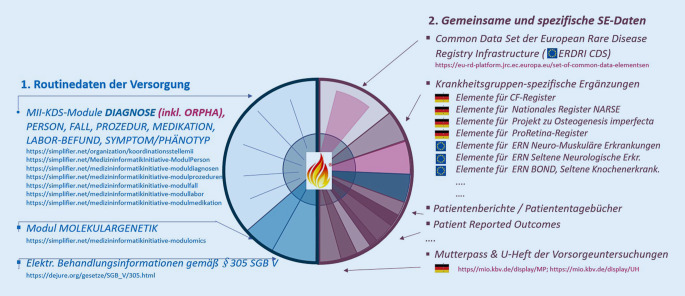

Abb. 2 skizziert das MII-Informationsmodell aus einer SE-spezifischen Perspektive. Sie zeigt eine blaue Seite der vorhandenen oder weitgehend SE-unabhängig im Aufbau befindlichen Module des MII-KDS. Der rote Halbkreis auf der rechten Seite repräsentiert die Datengestaltung nach den Anforderungen des NAMSE und die Vorarbeiten in Europa. Hier finden sich der gemeinsame Kern aller Register des ERN (European Reference Network), das „ERDRI CDS“ (Common Data Set der European Rare Disease Registration Infrastructure; [18]), ergänzende Komponenten einzelner ERN-Register und spezifische Bausteine für deutsche Register. Auch Patientendokumentationen werden hier genannt sowie die medizinischen Informationsobjekte „Mutterpass“ und „U-Heft“ der Kassenärztlichen Bundesvereinigung (KBV MIO; [16]).

Entgegen der grafischen Darstellung in Abb. 2 können die SE-Daten nicht strikt getrennt werden. Beispielsweise enthalten der molekulargenetische Befundbericht und das ERDRI CDS mehr oder weniger übereinstimmend Diagnoseangaben mit ICD- und Orpha-Codes, genetische Angaben gemäß Nomenklaturstandards HGVS (Human Genome Variation Society) und HGNC (Human Genome Nomenclature), phänotypische Angaben gemäß der Human Phenotype Ontology (HPO), Eigen- und Familienanamnese sowie Vitalparameter. Und auch das U‑Heft auf der roten Seite könnte gerade so gut auf der blauen Seite als Standardkomponente der Neugeborenendokumentation im Krankenhaus erscheinen. Die Vorschläge für die präzise, standardisierte Phänotypisierung werden in Abstimmung mit der Global Alliance for Genomics and Health (GA4GH) entwickelt [19]. Öffentlich aufbereitet, abgestimmt und publiziert wird der SE-erweiterte MII-Kerndatensatz im „FHIR Implementation Guide Rare Disease Documentation“ [20].

Die aufeinander abgestimmten Informationsmodelle der International Patient Summary (IPS; [21]), der Kassenärztlichen Bundesvereinigung (KBV MIO; [16]), der gematik (ISiK; [17]) und der MII (MII-FHIR-KDS) beruhen alle auf dem FHIR-Datenaustauschformat der Standardisierungsorganisation Health Level Seven (HL7) International [22].

Parallel zur Entwicklung der nächsten Generation von Routinedaten behält übrigens die Sekundärnutzung der Abrechnungsdaten als klassische Routinedaten ihre Bedeutung und gewinnt durch die Weiterentwicklung der „Datentransparenzstelle“ des Deutschen Instituts für Medizinische Dokumentation und Information (DIMDI) zum „Forschungsdatenzentrum im Bundesinstitut für Arzneimittel und Medizinprodukte“ (FDZ BfArM; [23]) einen neuen Schwung. Bedacht werden muss, dass die in der MI-Initiative in den Universitätskliniken weiterentwickelte nächste Generation von Routinedaten zunächst nur kurze Versorgungsphasen – nämlich die in den Versorgungsstellen der Universitätskliniken – widerspiegeln und dadurch im Prinzip nur den Charakter von Querschnittdaten haben. Für Längsschnittbetrachtungen und Verlaufsbeobachtungen wird man die neuen Routinedaten mit den klassischen verbinden, ebenso wie dies mit den vom BMBF angesprochenen Daten aus der Grundlagen- und sonstigen Forschung für andere Ergänzungen angestrebt wird.

## IT-basierte Unterstützung bei Diagnose und Therapiewahl

Mit den neuen Routinedaten werden in der MII und in angeschlossenen Vorhaben verschiedene Anwendungsszenarien der klinischen Forschung (inklusive kontrollierter prospektiver Studien), des Qualitätsmanagements, der Epidemiologie, der Versorgungsforschung und weiterer Felder entwickelt. Ein dringliches und zugleich herausforderndes Thema ist darunter die „IT-basierte Unterstützung von Diagnose und Therapiewahl bei seltenen Erkrankungen“, die das BMBF bei der Bekanntgabe des Förderkonzepts zur Medizininformatik im Jahr 2015 als Use Case vorgeschlagen hat.

Die direkteste Form der digitalen Unterstützung von Diagnose und Therapiewahl sind sogenannte Clinical Decision Support Systems (CDSS) oder als einfachere Hinweis-Apps sogenannte Symptom-Checker. Das Vorhaben CORD-MI entwickelt solche Systeme zwar nicht, es sollen aber an mehreren Standorten abgestimmte Erprobungen auf der Basis der standardisierten Routinedaten stattfinden. Dabei werden Synergien gehoben, indem die CORD-MI-Standorte Berlin, Göttingen, Erlangen und Freiburg und potenziell weitere sich am europäischen HORIZON-2020-Projekt „Screen4Care“ [24] beteiligen, dessen Zielsetzung die Verkürzung des oft langen Weges zur Diagnose mit digitalen Lösungen ist. Ein anderes Beispiel ist die Entwicklung eines „smarten Arztportals“ unter Beteiligung der CORD-MI-Partner Dresden [25] und Frankfurt [26] im Rahmen des Projektes *SATURN* [27]. Auch hier sollen auf der Basis von künstlicher Intelligenz (KI) für Betroffene mit unklarer Erkrankung die Diagnosezeit und die Suche nach den entsprechenden Expertinnen und Experten verkürzt werden. Ebenso werden im Vorhaben „Case Analysis and Decision Support“ (CADS) des Berlin Institute of Health (BIH) at Charité die Fallbeschreibungen mit Unterstützung durch das Werkzeug „SAMS“^9^ [5] standardisiert und maschinell ausgewertet.

Die zentralen Arbeitshypothesen von CORD-MI hierzu lauten, dass die einrichtungsübergreifende Nutzung von KI die semantische und syntaktische Interoperabilität der Datenbestände voraussetzt [28] und dass durch die einheitliche, genaue Beschreibung der Patientenmerkmale in den Routinedaten der positive Vorhersagewert (Verdacht auf Krankheit) und der negative Vorhersagewert (Ausschluss von Krankheiten) erhöht werden können. So kann gerade bei den SE eine schnelle Diagnose zu einer frühen Therapie führen und damit Folgeschäden vermeiden. Als Beispiel sei die Seltene Erkrankung „spinale Muskelatrophie“ genannt, die in den Katalog des Neugeborenen-Screenings aufgenommen wurde: da hier die frühe Gentherapie mit dem Wirkstoff Onasemnogen-Abeparvovec den Muskelschwund bremsen kann und Heilung bereits durch einmalige Gabe verspricht.

Für die kommenden Jahre wird die Entwicklung vieler neuer Gentherapien und Enzymersatztherapien erwartet. Durch die neuen Routinedaten soll in erster Linie die Diagnose beschleunigt werden. Der Einsatz von KI ist dabei nur eine Komponente. Das Zusammentreffen von natürlicher Intelligenz in Fallkonferenzen kann (noch) nicht ersetzt, wohl aber durch Register mit standardisierter Datenaufbereitung unterstützt werden. Das mit CORD-MI assoziierte Nationale Register für (Ultra‑)Seltene Erkrankungen (NARSE) wird speziell auf Erkrankungen ausgerichtet, bei denen neue Therapien erwartet werden. Auch in verschiedenen Registern für Patienten mit unklarer Diagnose wird eine standardisierte Beschreibung von Symptomen und Phänotypen angestrebt, um den notwendigen Austausch in Fallkonferenzen zu unterstützen. Ergänzend wird geprüft werden, inwieweit durch die neuen Routinedaten auch prospektive Therapiestudien und Pharmakovigilanz effizienter gestaltet werden können.

## Digitale Vernetzung der Kliniken und Unterstützung der Forschung

**Figure 3:**
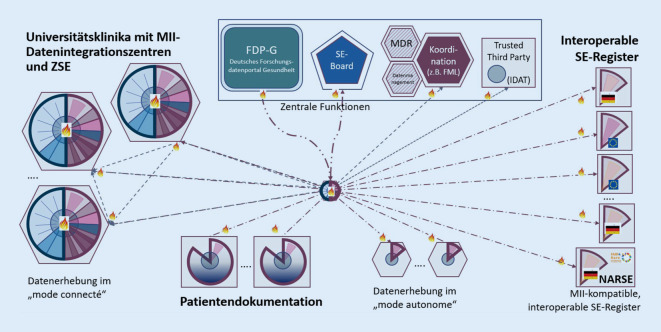

Im digital vernetzten, weiterhin föderierten Gesundheitswesen behalten die Krankenhäuser einschließlich der Universitätskliniken gemäß dem MII-Konzept die Verantwortung für ihre lokalen Gesundheits- und genetischen Daten und die Hoheit darüber. Sie stimmen sich lediglich im Hinblick auf semantische und syntaktische Interoperabilität in ihren DIZ miteinander ab. Den NAMSE-Anforderungen folgend sind die Diagnosen mit Orpha-Kennnummern kodiert, alle klinischen Befunde sind strukturiert digitalisiert und die genomischen Befunde sind mit standardisierten phänotypischen Beschreibungen verknüpft (vgl. Beitrag zu Datenstandards für Seltene Erkrankungen von Robinson und Graessner in diesem Themenheft). Diese Konstellation wird für die repräsentativen Klinikstandorte („Universitätsklinika mit MII-Datenintegrationszentren [DIZ] und Zentren für Seltene Erkrankungen [ZSE]“) auf der linken Seite von Abb. 3 skizziert.

Antragstellende Forschende der ZSE, Patientengruppen und externe Wissenschaftlerinnen und Wissenschaftler können sich mit Forschungsfragen an das Deutsche Forschungsdatenportal für Gesundheit (FDP-G) wenden (Abb. 3, Mitte). Die angefragten Standorte können prüfen, ob die Anfragen zu klinischen Studien zu ihrem Versorgungsspektrum passen (= Machbarkeitsanalysen), und können gegebenenfalls anbieten, ergänzende Studienteilnehmer anzusprechen (= Probandenrekrutierung). Mit den Studienkollektiven, die beispielsweise Betroffene mit definierten Erkrankungen in verschiedenen Einrichtungen umfassen, können sowohl retrospektive Beobachtungsstudien als auch prospektive, randomisiert kontrollierte klinische Studien (RCT) realisiert werden. Für die RCT müssen die Routinedaten durch eine Protokolldatei ergänzt werden. Für gemeinsame Auswertungen müssen die Daten die geschützten Räume der DIZ nicht verlassen. In konsiliarischen SE-Boards der Arbeitsgemeinschaft der Zentren für Seltene Erkrankungen (AG ZSE) können einzelne Fälle vergleichend besprochen werden, sofern die jeweiligen Patientinnen und Patienten hierin eingewilligt haben.

Es wird angestrebt, dass medizinische Register und Patientendokumentationen (Abb. 3, rechts und unten) in gleicher Weise an das FDP‑G und die SE-Board-Funktionalität angeschlossen werden wie die Repositorien in den DIZ (beginnend mit Registern in den Standorten). Dadurch können diese auch in die einrichtungsübergreifenden, datenschutzkonformen Auswertungsmodelle der MII einbezogen werden.

Neben vielen künftigen Möglichkeiten, die die digitale Zusammenarbeit für die SE mit sich bringt, sei abschließend ein Beispiel unter vielen aus den aktuellen CORD-Aktivitäten genannt: Für Schwangerschaften bei Frauen, die an zystischer Fibrose (CF) erkrankt sind, gibt es eine Leitlinie, die die Entbindung in einem Fachzentrum vorsieht. Anhand einer Studie wird momentan überprüft, wie viele Geburten von Patientinnen mit CF in den beteiligten Universitätskliniken erfasst wurden, d. h. wirklich in einem spezialisierten Fachzentrum stattfanden, und welche Komplikationen bei den Geburten dokumentiert wurden. Zum einen wird damit getestet, inwiefern es auf technischer Seite wirklich möglich ist, aus den diversen Krankenhaussystemen die verschiedenen Daten zusammenzuführen, und ob die resultierenden Ergebnisse mit den Einschätzungen der behandelnden Ärztinnen und Ärzte zu ihren Patientinnenzahlen kongruent sind. Zum anderen könnten aus der Diskussion der Ergebnisse auch Strategien entwickelt werden, wie womöglich mehr Schwangere mit CF gezielt den spezialisierten Fachzentren zugeführt und mögliche Schädigungen der Neugeborenen verhindert werden können.^10^

## Fazit und Ausblick

Das Vorhaben CORD-MI ist durch die im Herbst 2022 in Kooperation mit dem FDP‑G laufenden „CORD-MI Starterstudien“ im Begriff zu belegen, dass die digitale Zusammenarbeit der Universitätskliniken im Rahmen der Medizininformatik die gemeinsame Datennutzung für Menschen mit SE im Prinzip ermöglicht.

In der Broschüre zum Förderkonzept Medizininformatik hieß es in 2015 vom BMBF: „Das digital vernetzte Gesundheitswesen ist zurzeit noch eine Zukunftsvision. Durch Fortschritte in der Medizininformatik ist es aber möglich, dieser Vision einen großen Schritt näher zu kommen.“ Die Beteiligten des Vorhabens CORD-MI vertrauen auf den Bestand der Datenintegrationszentren und des Deutschen Forschungsdatenportals für Gesundheit sowie deren fortgesetztes Engagement für die SE. Die „CORDistas“ sehen sich selbst in der Pflicht, mit den Kernelementen der MI-Initiative unter Beachtung der NAMSE- und der EU-Anforderungen die digitale Vernetzung der Zentren für Seltene Erkrankungen und Dokumentationsfortschritte weiter voranzubringen. Hierfür sollen in nachfolgenden Projekten sowohl die digitalen Versorgungsakten als auch ausgewählte deutsche und europäische Register für Seltene Erkrankungen als auch spezifische Dokumentationen von Selbsthilfe- und Betroffenengruppen einbezogen werden. Vorrangig zu nennende Nachfolgeprojekte von CORD-MI sind der Aufbau des föderierten Nationalen Registers für Seltene Erkrankungen (NARSE) und das EU-Vorhaben „Screen4Care for Shortening the Path to Rare Disease Diagnosis by Digital Technologies“ mit bleibender enger Anbindung an die MI-Initiative.

## Footnotes

1 Als „Seltene Erkrankungen“ (SE) gelten in der Europäischen Union (EU) Krankheiten, von denen nicht mehr als 5 von 10.000 Menschen betroffen sind.

2 TRANSLATE-NAMSE: Projekt zur Verbesserung der Versorgung von Menschen mit Seltenen Erkrankungen.

3 ZSE-DUO-Projekt: duale Lotsenstruktur zur Abklärung unklarer Diagnosen in Zentren für Seltene Erkrankungen.

4 Phenopacket: standardisierte Datenpakete zur Erstellung von Krankheitsmodellen, in denen Phänotypbeschreibungen mit Krankheits‑, Patienten- und genetischen Informationen verknüpft werden.

5 ICD (Internationale statistische Klassifikation der Krankheiten und verwandter Gesundheitsprobleme).

6 OPS (Operationen- und Prozedurenschlüssel).

7 Orpha-Kennnummer („OrphaCode“): Kennnummer für einzelne Seltene Erkrankungen, zu finden in der Datenbank „Orphanet“.

8 SNOMED CT (Systematized Nomenclature of Human and Veterinary Medicine Clinical Terms): Nomenklatur zur Indexierung medizinischer Sachverhalte.

9 SAMS (Symptom Annotation Made Simple): Datenbank und Phänotypisierungswerkzeug.

10 Weitere und vertiefende Beispiele der Nutzung von Routinedaten finden sich in einer längeren Fassung des vorliegenden Aufsatzes, die bei den Autoren angefragt werden kann.
